# Can Light Physical Activity Improve Cognition Among Older Adults? A Scoping Review

**DOI:** 10.1093/geroni/igaa057.3288

**Published:** 2020-12-16

**Authors:** Emily Erlenbach, Edward McAuley, Neha Gothe

**Affiliations:** University of Illinois at Urbana-Champaign, Urbana, Illinois, United States

## Abstract

Although the physical and cognitive benefits of moderate-vigorous intensity physical activity (MVPA) for older adults is well documented, this population often faces age-related functional and physical limitations impeding recommended MVPA participation. Recently, there has been a surge of interest in the independent health benefits of light-intensity physical activity (LPA) and its association with morbidity and mortality risk. LPA is the most common form of activity among older adults and its potential to combat cognitive aging needs to be investigated. The purpose of this scoping review was to catalog existing evidence on the association between device-based or technologically measured LPA and cognition among healthy older adults, identify trends in the literature, and pinpoint future areas of research. Six electronic databases were searched between January and August 2020. Eighteen published studies met the inclusion criteria: one acute exercise study, two randomized control trials (RCTs), twelve cross-sectional studies, and three longitudinal studies. Overall, n=9 studies (n=1 RCT, n=7 cross-sectional, and n=1 longitudinal) reported a significant, positive relationship between LPA and one or more cognitive outcomes including memory, attention, executive function and global cognition (MMSE/MOCA). These heterogeneous findings can largely be attributed to the diverse study designs, inconsistent definitions of LPA and numerous assessments used to test the cognitive domains. Collectively, these findings suggest LPA may be a potential lifestyle intervention to improve cognition among healthy older adults. However, the inconsistent approaches used among these studies suggests a more concerted, unified scientific approach and rigorous methodology are needed to further understand the LPA-cognition relationship.

